# Supplementary figures and images for: Pathobiology and dysbiosis of the respiratory and intestinal microbiota in 14 months old Golden Syrian hamsters infected with SARS-CoV-2
Source: PLoS Pathog. 2022 Oct 24;18(10):e1010734. doi: 10.1371/journal.ppat.1010734 (PMC9632924; doi:10.1371/journal.ppat.1010734)

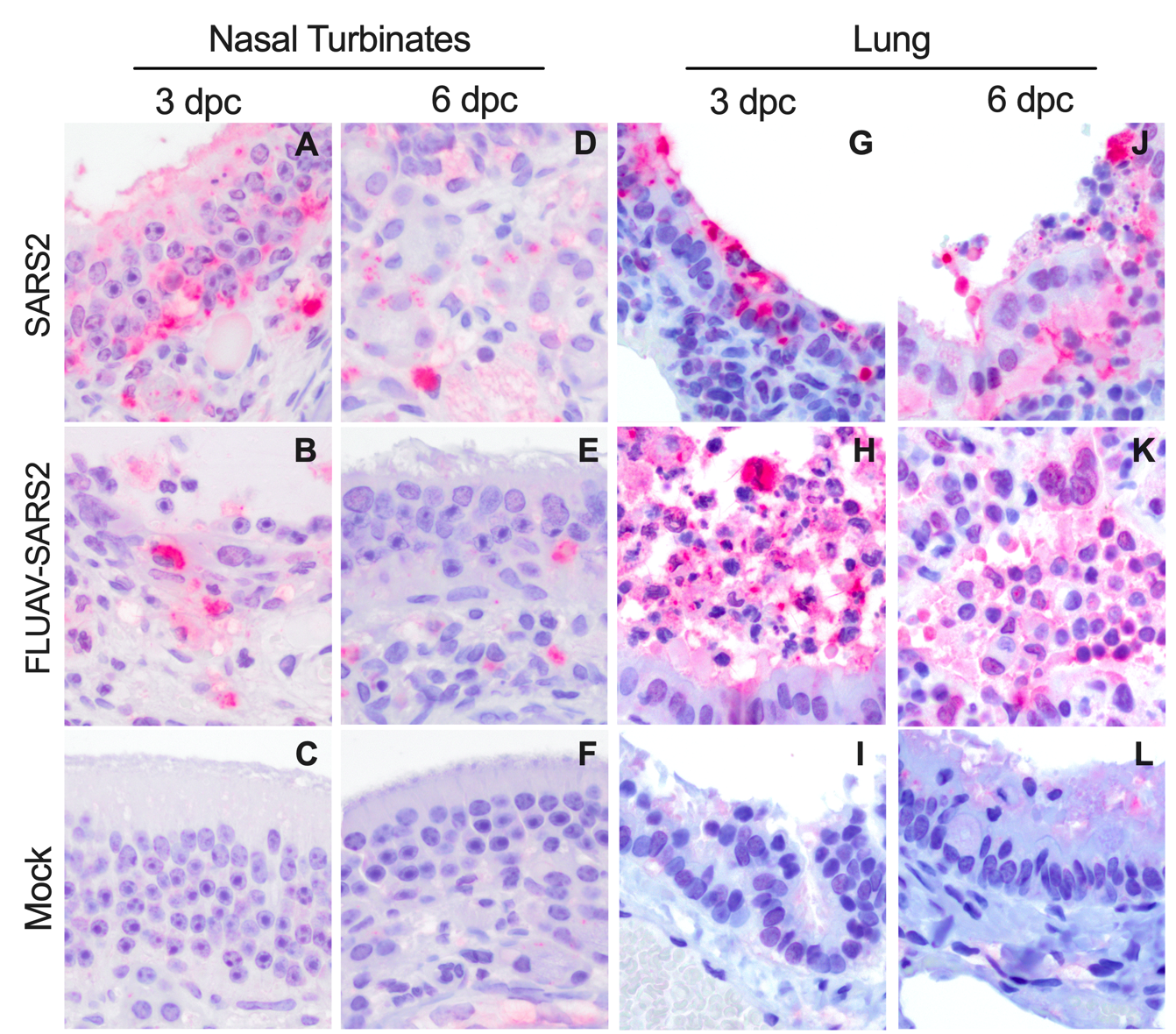

Supplement: S1 Fig — (A-F) Nasal turbinates. (G-L) Lungs. (A-B) Variable amounts of intracytoplasmic virus antigen (red) immunolabeling is present in epithelial cells in challenged GSH. (D-E) Low levels of virus antigen are present in the epithelial cells as evidenced by faint red immunostaining. (C, F) No significant virus antigens are observed in mock hamsters’ nasal turbinates. (G-H) Moderate amounts of virus antigen (red) are present in bronchiolar epithelium and intraluminal necrotic cellular debris in challenged GSH. (J-K) The intracytoplasmic virus antigen (red) is scarce at this timepoint. (I, L) No significant amount of virus antigen (red) immunolabeling are observed in the mock hamsters’ lungs. All immunohistochemistry images are at 40X magnification. (TIF) [file ppat.1010734.s001.tif]

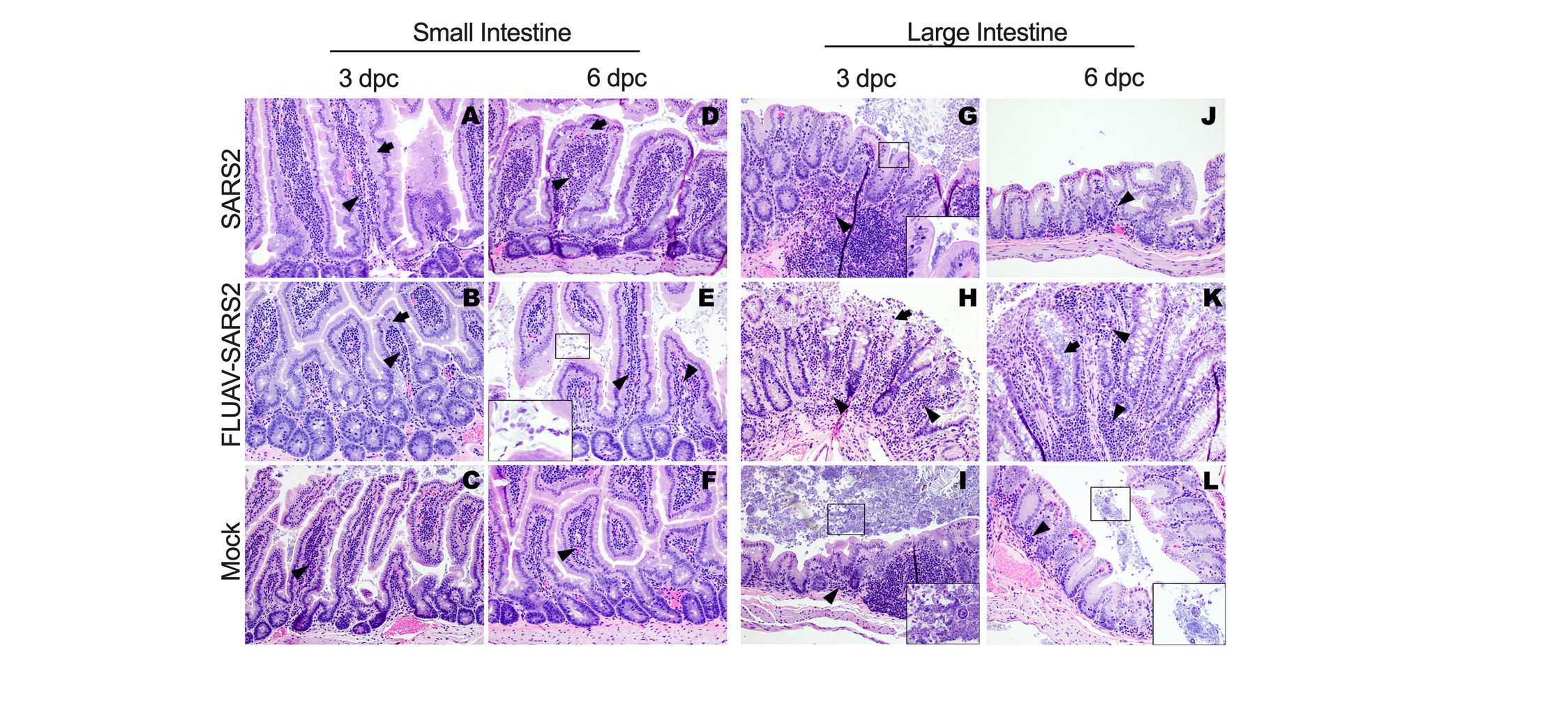

Supplement: S2 Fig — (A-F) Small numbers of eosinophils, lymphocytes and macrophages are expanding the villi lamina propria (arrowheads) regardless of the timepoint and treatment groups. In the infected groups, the lacteals are dilated by small amounts of eosinophilic fluid (arrows). Increased numbers of protozoa compatible with Giardia spp. (insert) are populating the lumina in most sections. (G-H) At 3 dpc, moderate numbers of eosinophils, macrophages, and lymphocytes (arrowheads) are expanding the mucosa lamina propria in infected hamsters. The surface enterocytes are necrotic and sloughing into the lumen (arrow). The lumina contains mixed bacteria (insert) and protozoa. (I) In the mock infected hamsters, only small clusters of eosinophils are infiltrating the mucosa (arrowhead). Rare round protozoa (insert) are compatible with Entamoeba spp. (J and L) Small numbers of neutrophils infiltrate the mucosa lamina propria (arrowhead) in SARS2-only infected hamsters and mocks. Intestinal lumina contain mixed bacteria and a few round protozoa (insert) compatible with Entamoeba spp. (K) In FLUAV-SARS2 hamsters at 6 dpc, lesions recapitulate what is described at 3 dpc. The mucosa is infiltrated by mixed inflammatory cells (arrowheads), the glands lumen is dilated by increased bacteria (arrow), and enterocytes are sloughing into the lumen. All H&E images are pictured at 20X and inserts are at 40X magnification. (TIF) [file ppat.1010734.s002.tif]

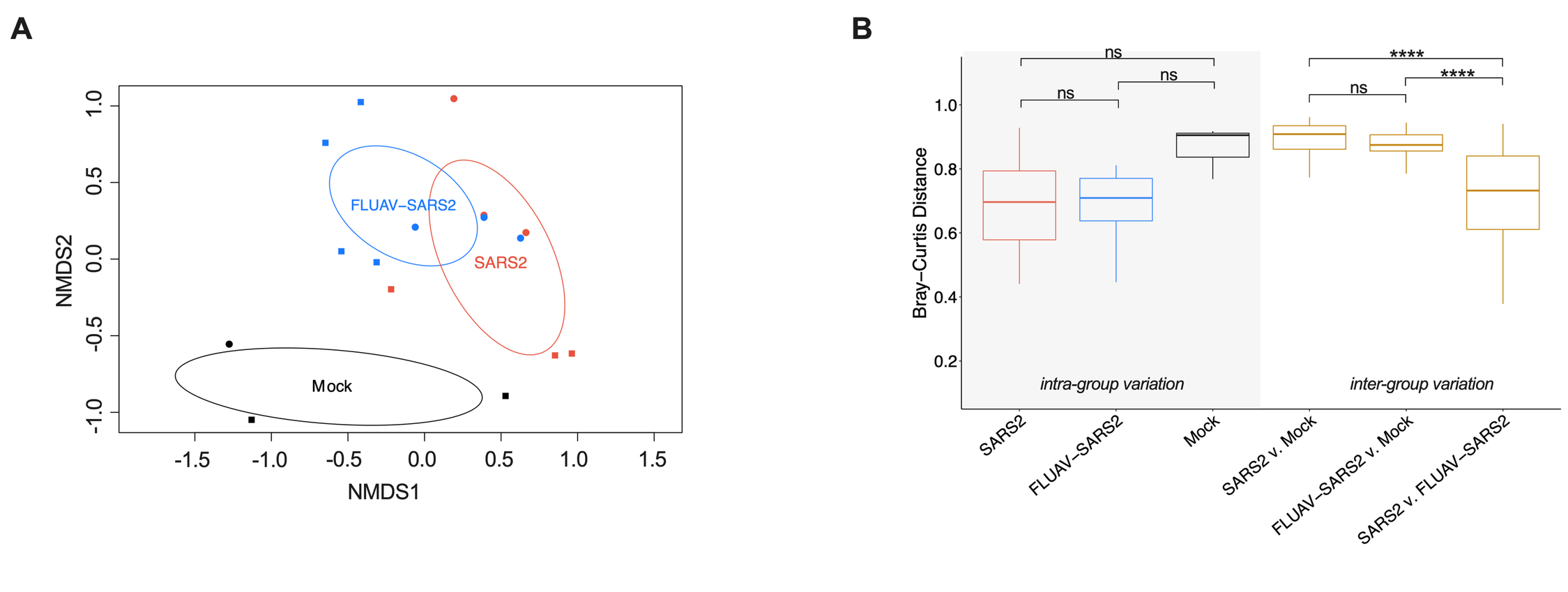

Supplement: S3 Fig — (A) NMDS plot of weighted Bray-Curtis dissimilarity distance of the lung samples. The color designates groups (SARS2: red, FLUAV-SARS2: blue, and mock: white) and dpc by the shape (3 dpc, circle and 6 dpc: square). Ellipses were constructed to include all points within the group. (B) Comparison of weighted Bray-Curtis dissimilarity distance within each group and across multiple comparisons of the lung samples. Intra-group variation is marked grey shading, while the inter-group comparisons are marked gold. Pair-wise comparisons were performed using the Wilcox test with Bonferroni correction. (TIF) [file ppat.1010734.s003.tif]

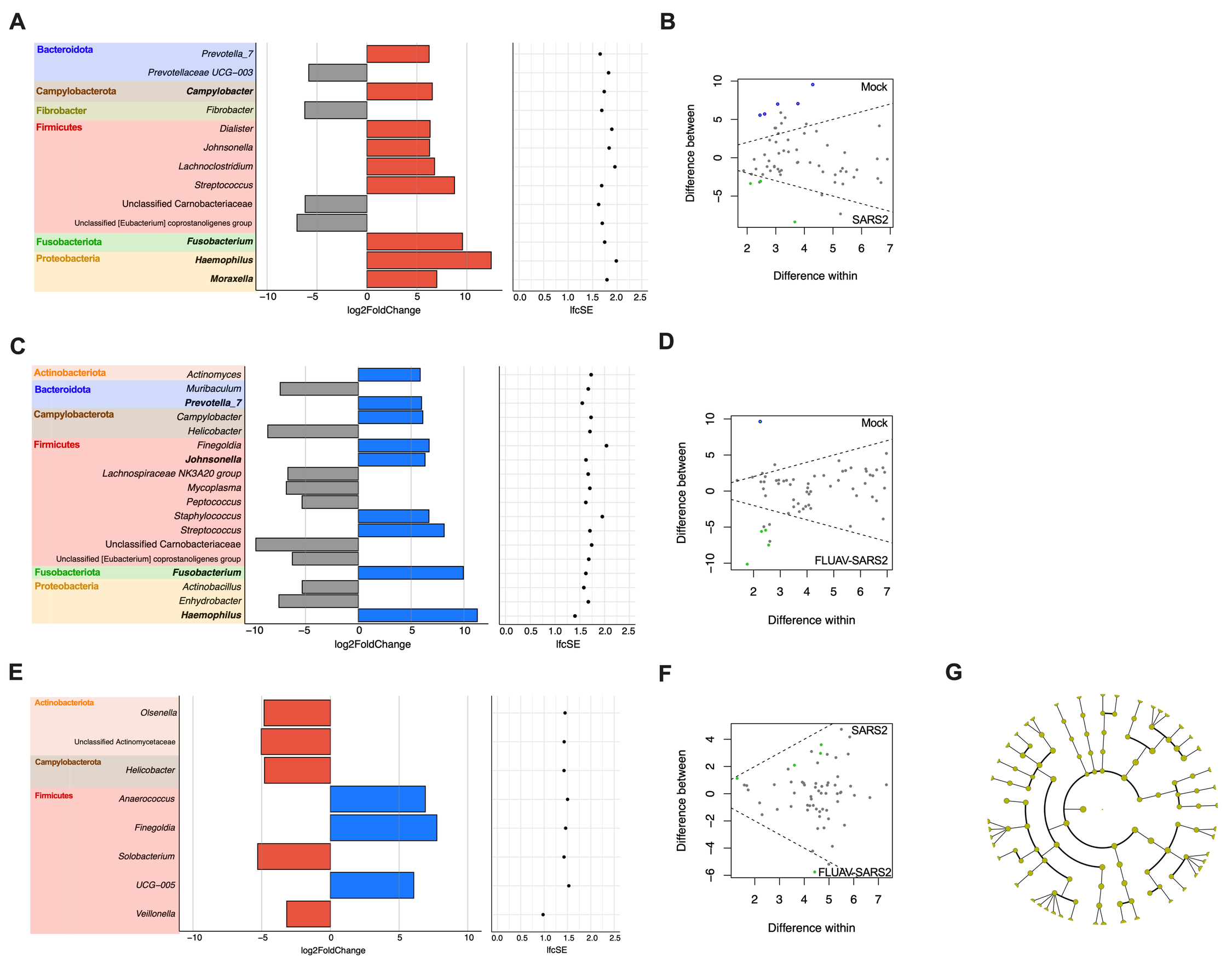

Supplement: S4 Fig — (A, C, E) Bacterial taxa identified at different relative abundances between (A) SARS2 (red), and mock (grey) or (C) FLUAV-SARS2 (blue), and mock (grey) or (E) SARS2 (red) and FLUAV-SARS2 (blue) groups using DeSeq2. Significantly enriched taxa were plotted with log2 fold change and the corresponding standard error estimate. (B, D, F) Effect size plot showing the median log2 fold difference (difference between) by the median log2 dispersion (difference within) when comparing (B) SARS2 and mock or (D) FLUAV-SARS2 and mock or (F) SARS2 and FLUAV-SARS2. Taxa considered significant by the Wilcox test are shown in green, taxa with BH corrected p-values are shown in red, while taxa with an effect size greater than 1.5 are outlined in blue. (G) Cladogram showing significantly (p<0.05) abundant taxa between SARS2 (red) and FLUAV-SARS2 (blue) using linear discriminant analysis effect size (LEfSe) analysis with default parameters. Taxa are color-coded by phylum, and bold taxa are considered significant in at least 2 of the 3 differential analyses performed (Deseq2, ALDEx2, and LefSE). Significant taxa using Deseq2 were determined by having a p-value < 0.01, while significant taxa using ALDEx2 and LEfSE were determined by having a p-value < 0.05. (TIF) [file ppat.1010734.s004.tif]

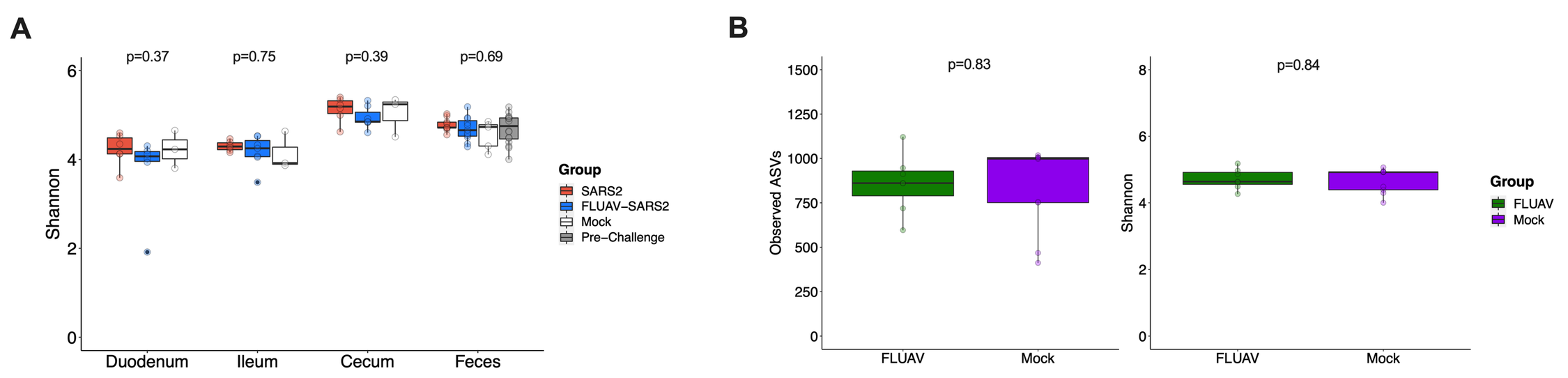

Supplement: S5 Fig — (A) Shannon diversity of the intestinal/fecal samples between FLUAV-SARS2 (red), SARS2 (blue), mock (white), and pre-challenge (grey) containing all dpc from the rarified ASV count table. Multiple group comparisons were performed using Kruskal-Wallis, while pair-wise comparisons were conducted using the Wilcox test with Bonferroni correction. (B) Alpha diversity measure of the observed number of ASVs (left) and Shannon diversity (right) between FLUAV exposed (green) and mock (purple) pre-challenge fecal samples from the rarified ASV count table. Pair-wise comparisons were conducted using the Wilcox test with Bonferroni correction. (TIF) [file ppat.1010734.s005.tif]

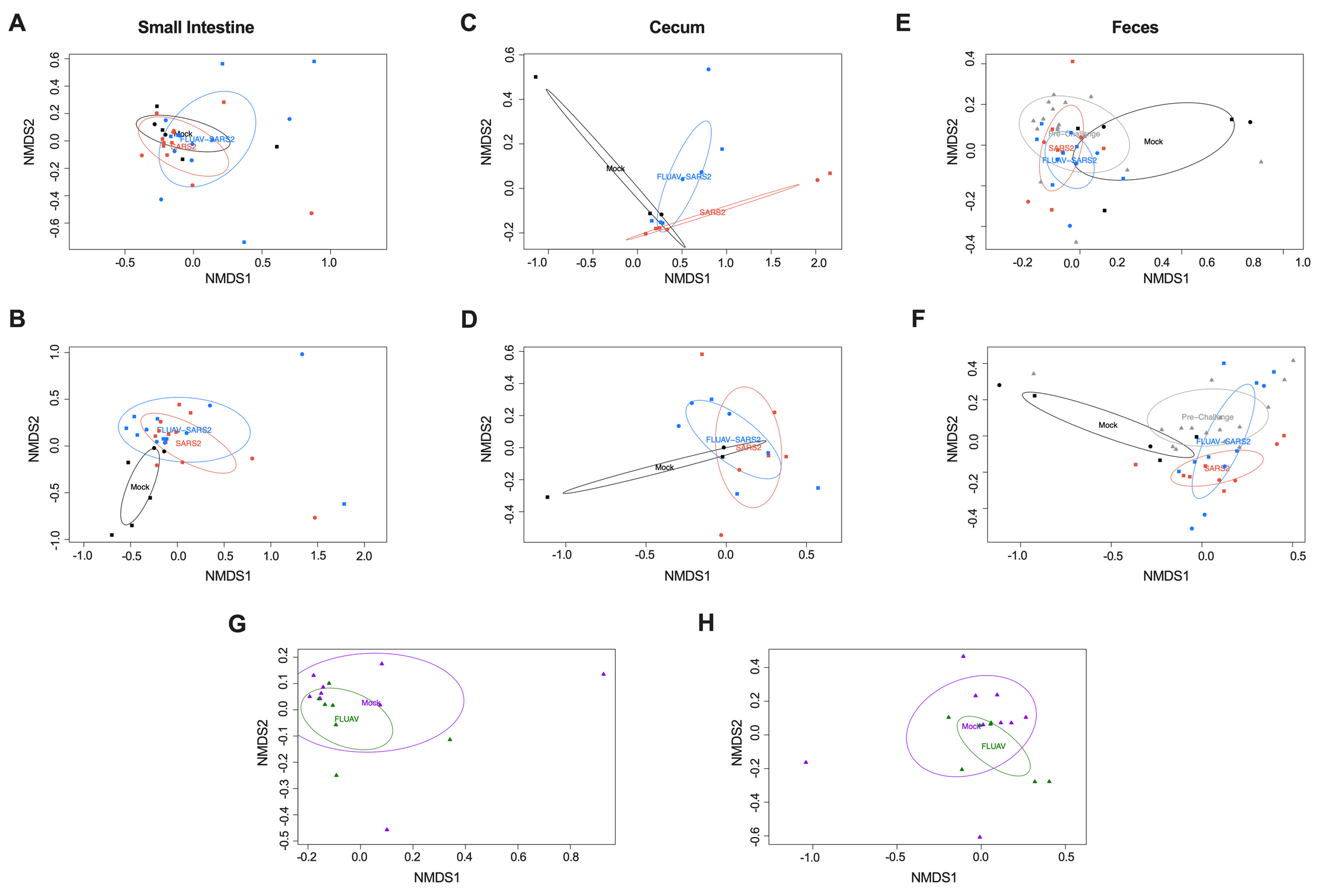

Supplement: S6 Fig — NMDS plot of unweighted Jaccard distance (A, C, E, G) and weighted Bray-Curtis (B, C, F, H) dissimilarity distance of the small intestine, including the duodenum and ileum (A and B), cecum (C and D) and the feces (E and F). Groups are designated by the color (FLUAV-SARS2: red, SARS2: blue, mock: white, pre-challenge: grey) and sample type by the shape (duodenum: circle and ileum: square) or dpc by the shape (3 dpc, circle and 6 dpc: square). Ellipses were constructed using the standard deviation. Comparison between FLUAV exposure and mock controls when analyzing pre-challenge fecal samples were also compared (G and H). (TIF) [file ppat.1010734.s006.tif]

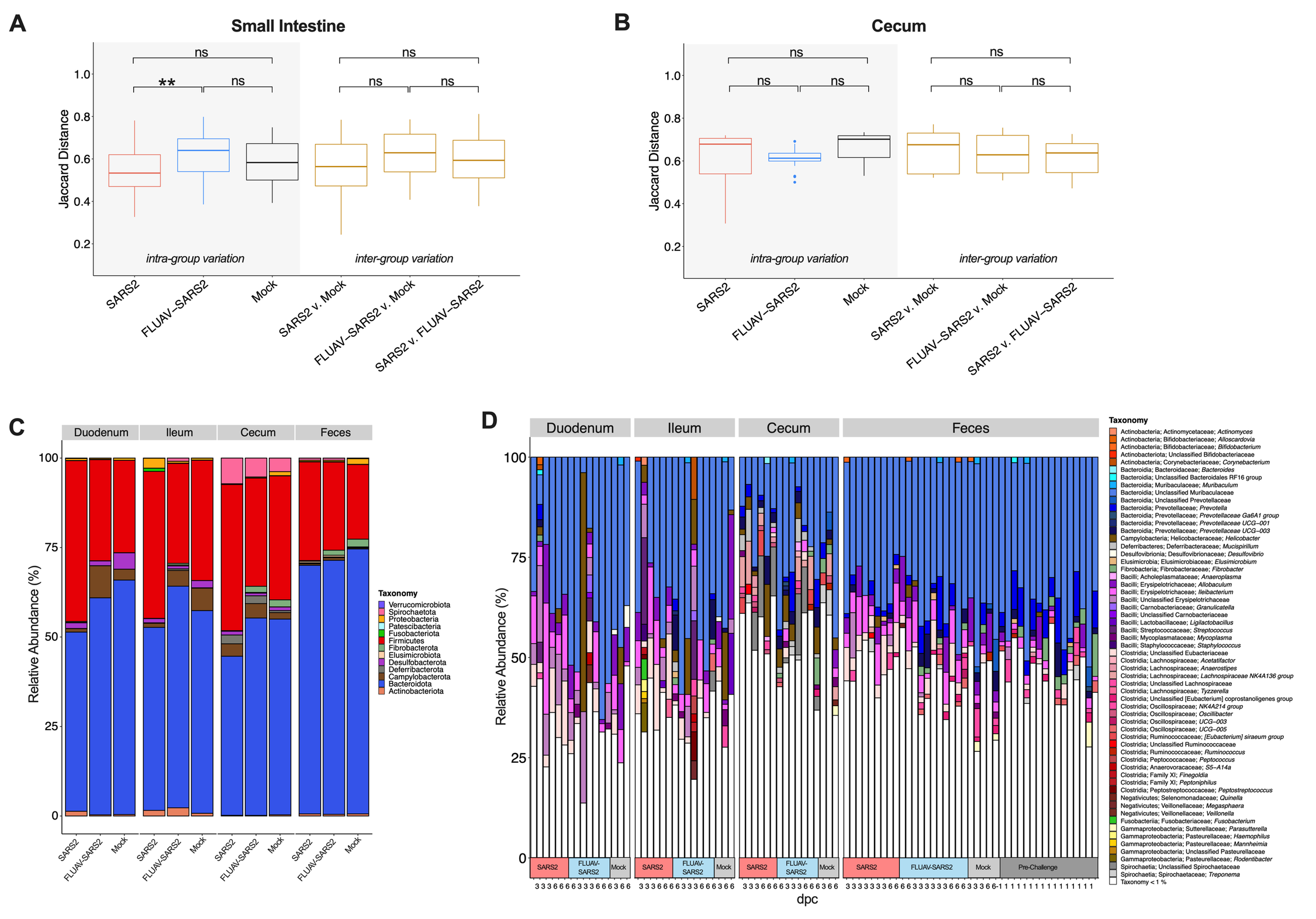

Supplement: S7 Fig — Comparison of unweighted Jaccard dissimilarity distance within each group and across multiple comparisons in the small intestine, including the duodenum and ileum (A) and the cecum (B). Intra-group variation is marked with grey shading, while the inter-group comparisons are colored gold. Pair-wise comparisons were performed using the Wilcox test with Bonferroni correction. (C) Relative abundances agglomerated at the phylum level separated by sample type and group. (D) Relative abundances of each hamster at the lowest taxonomic rank identified separated by sample type, group, and ordered by dpc. (TIF) [file ppat.1010734.s007.tif]

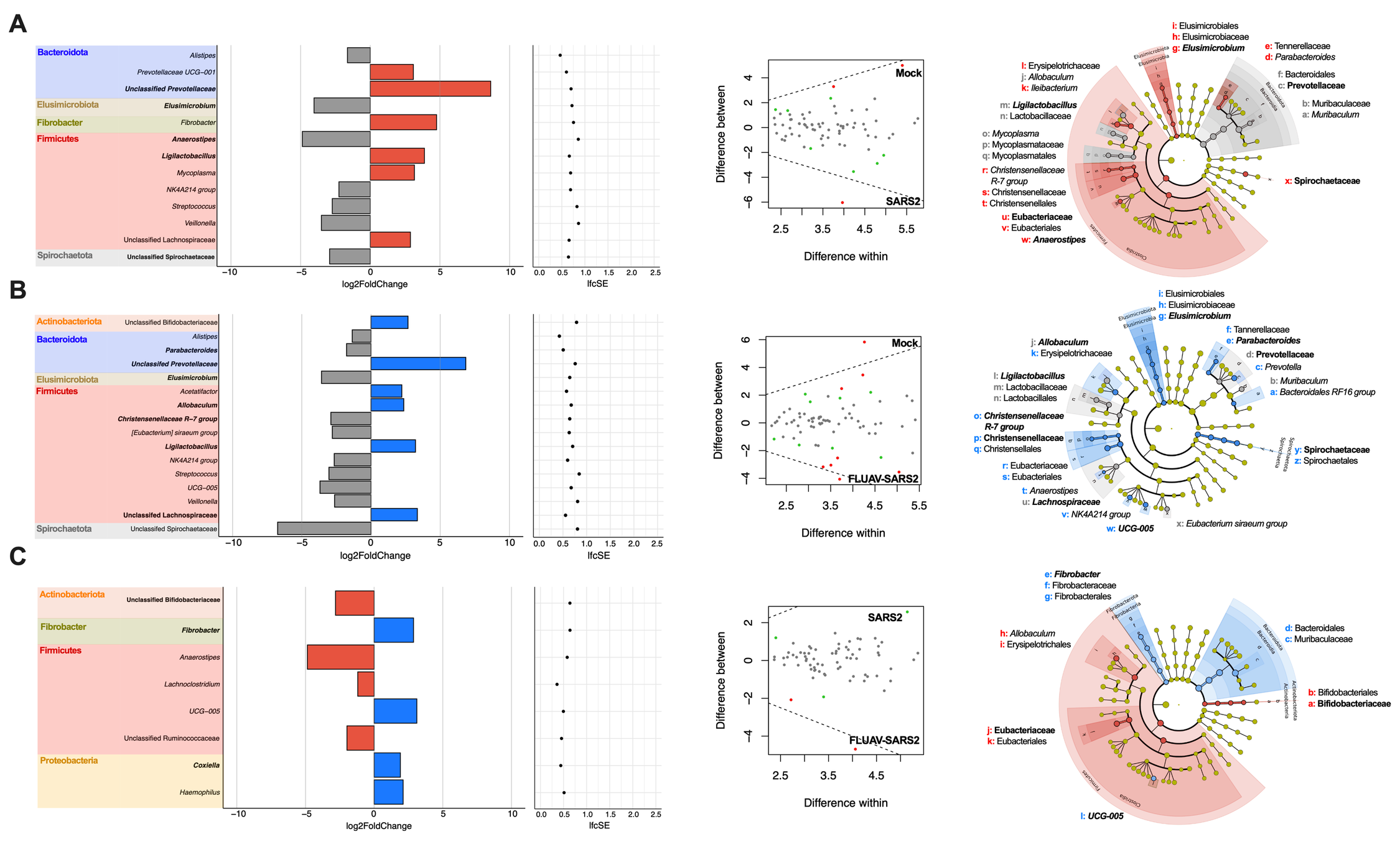

Supplement: S8 Fig — Bacterial taxa identified at different relative abundances between (A left) SARS2 (red), and mock (grey) or (B left) FLUAV-SARS2 (blue), and mock (grey) or (C left) SARS2 (red) and FLUAV-SARS2 (blue) groups using DeSeq2 when all sample types (duodenum, ileum, cecum, and feces) were combined. Significantly enriched taxa were plotted with log2 fold change and the corresponding standard error estimate. Taxa in bold were those considered significant in at least 2 of the 3 differential analyses performed. Effect size plot showing the median log2 fold difference (difference between) by the median log2 dispersion (difference within) when comparing (A middle) SARS2, and mock or (B middle) FLUAV-SARS2, and mock or (C middle) SARS2 and FLUAV-SARS2 when all sample types (duodenum, ileum, cecum, and feces) were combined. Taxa considered significant by the Wilcox test are shown in green, taxa considered significant by BH corrected p-values are shown in red, while taxa with an effect size greater than 1.5 are outlined in blue. Cladogram showing significantly (p<0.05) abundant taxa between (A right) SARS2 (red) and mock (grey) or (B right) FLUAV-SARS2 (blue), and mock (grey) or (C right) SARS2 (red) and FLUAV-SARS2 (blue) using linear discriminant analysis effect size (LEfSe) analysis with default parameters when all sample types (duodenum, ileum, cecum, and feces) were combined. Taxa are color-coded by phylum, and bold taxa are considered significant in at least 2 of the 3 differential analyses performed. Significant taxa using Deseq2 were determined by having a p-value < 0.01, while significant taxa using ALDEx2 and LEfSE were determined by having a p-value < 0.05. (TIF) [file ppat.1010734.s008.tif]

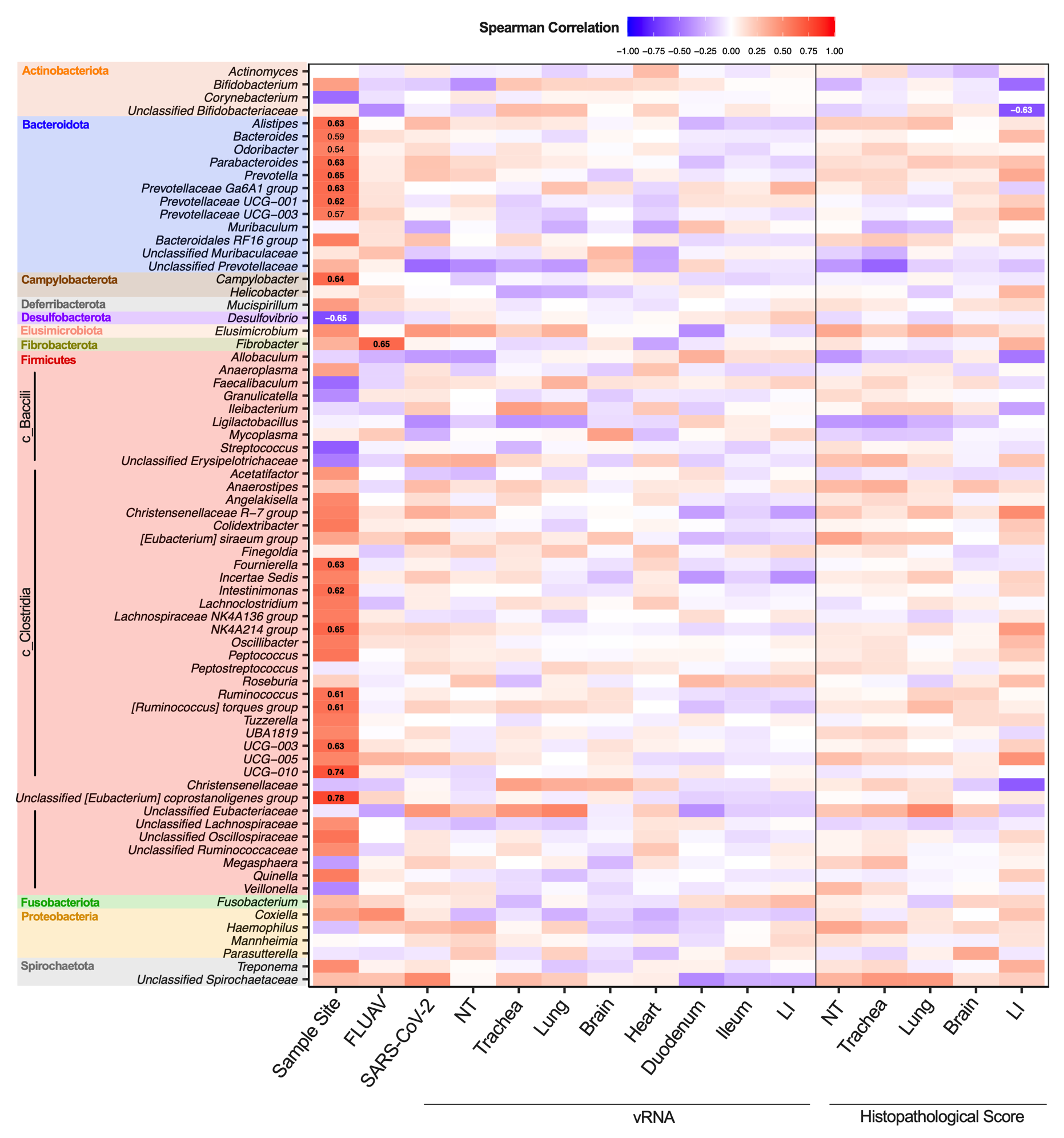

Supplement: S9 Fig — A heatmap illustrating the Spearman correlation among different infection factors and the relative abundance of bacterial taxa when all sample types (duodenum, ileum, cecum, and feces) were combined. Correlations with histopathological score in the heart and small intestine were not included. Pre-challenge feces were also not included in this analysis. Taxa are color-coded by phylum. Correlation labels have a spearman correlation value > 0.60 or < -0.60. FLUAV–inoculated with FLUAV and then challenged with SARS-CoV-2 (mock-challenge GSH were not included), SARSCoV2 –challenged with SARS-CoV-2, NT- nasal turbinates, LI–large intestine. (TIF) [file ppat.1010734.s009.tif]

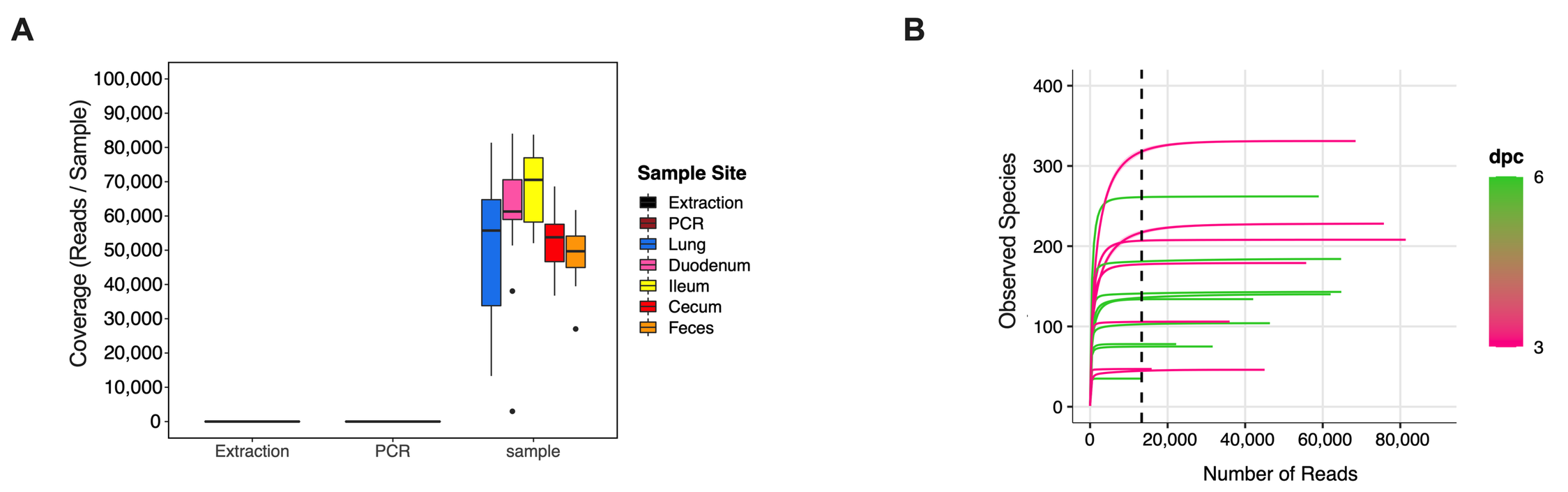

Supplement: S10 Fig — (A) Sample coverage measured by the number of reads per sample separated by controls (DNA extraction and PCR blanks) and sample. The samples are color-coded by sample type (Lung: blue, Duodenum: pink, Ileum: yellow, Cecum: red, and Feces: orange). (B) Rarefaction curve of the lung samples colored by dpc (3 dpc: pink, 6 dpc: green). The dashed line (x = 13,306) represents the number of reads each sample was rarified for downstream diversity analyses. (TIF) [file ppat.1010734.s010.tif]
