# Supplementary material for: Pathobiology and dysbiosis of the respiratory and intestinal microbiota in 14 months old Golden Syrian hamsters infected with SARS-CoV-2
Source: PLoS Pathog. 2022 Oct 24;18(10):e1010734. doi: 10.1371/journal.ppat.1010734 (PMC9632924; doi:10.1371/journal.ppat.1010734)
Supplement: S1 Table — Subjectively scored tissues (blind) based on percentage of the total parenchyma affected by lesions and inflammation as: none (0%), mild; <15% (1), mild to moderate; 16–30% (2), moderate; 31–50% (3), moderate to severe 51–75% (4) and severe; ≥75% (5). A dash is used to separate the score evaluated for each individual hamster separated by group and days post challenge (dpc). (DOCX) [file ppat.1010734.s011.docx]

**S1 Table.** **H&E** **pathology scores of different tissues.**

| **Group** | **dpc** | **Brain** | **Heart** | **Small Intestine** | **Cecum** |
| --- | --- | --- | --- | --- | --- |
| SARS2 | 3 | 0 / 0 / 0 | 0 / 0 / 0 | 1 / 1 / 1 | 1 / 2 / 1 |
|  | 6 | 0 / 0 / 1 | 0 / 0 / 0 | 1 / 1 / 1 | 1 / 2 / 1 |
| FLUAV-SARS2 | 3 | 1 / 0 / 0 | 0 / 0 / 0 | 1 / 1 / 1 | 3 / 1 / 2 |
|  | 6 | 0 / 0 / 0 / 0 | 0 / 0 / 0 / 0 | 1 / 1 / 1 /1 | 1 / 1 / 1 / 3 |
| Mock | 3 | 0 | 0 | 1 | 1 |
|  | 6 | 0 / 0 | 0 / 0 | 1 / 1 | 1 / 1 |

Subjectively scored tissues (blind) based on percentage of the total parenchyma affected by lesions and inflammation as: none (0%), mild; <15% (1), mild to moderate; 16-30% (2), moderate; 31-50% (3), moderate to severe 51-75% (4) and severe; ≥75% (5). A dash is used to separate the score evaluated for each individual hamster separated by group and days post challenge (dpc).
