# Supplementary material for: Pathobiology and dysbiosis of the respiratory and intestinal microbiota in 14 months old Golden Syrian hamsters infected with SARS-CoV-2
Source: PLoS Pathog. 2022 Oct 24;18(10):e1010734. doi: 10.1371/journal.ppat.1010734 (PMC9632924; doi:10.1371/journal.ppat.1010734)
Supplement: S2 Table — Shown in bold are enriched groups that were considered significant in at least 2 of the 3 differential analyses performed. Adjusted p-values are reported: Deseq2 (Benjamini-Hochberg adjusted p-value) and ALDEx2 (Benjamini-Hochberg adjusted p-value using Wilcox t-test). p < 0.05 was considered significant for ALDEx2 and LefSE analysis and p < 0.01 was considered significant for Deseq2 analysis. (DOCX) [file ppat.1010734.s012.docx]

|  | SARS2 v. Mock | | | | FLUAV-SARS2 v. Mock | | | | SARS2 v. FLUAV-SARS2 | | | |
| --- | --- | --- | --- | --- | --- | --- | --- | --- | --- | --- | --- | --- |
| **Genus** | *Deseq2* | *ALDEx2* | *LefSE* | *Enriched* | *Deseq2* | *ALDEx2* | *LefSE* | *Enriched* | *Deseq2* | *ALDEx2* | *LefSE* | *Enriched* |
| *Prevotella_7* | 0.00096 | 0.50 | > 0.05 | SARS2 | 0.00063 | 0.33 | 0.015 | **FLUAV-SARS2** | 0.97 | 0.99 | > 0.05 | NA |
| *Campylobacter* | 0.00096 | 0.51 | 0.043 | **SARS2** | 0.0019 | 0.48 | > 0.05 | FLUAV-SARS2 | 0.91 | 0.93 | > 0.05 | NA |
| *Streptococcus* | 2.98E-06 | 0.97 | > 0.05 | SARS2 | 2.34E-05 | 0.97 | 0.034 | **FLUAV-SARS2** | 0.79 | 0.97 | > 0.05 | NA |
| *Johnsonella* | 0.0026 | 0.67 | > 0.05 | SARS2 | 0.0063 | 0.37 | 0.015 | **FLUAV-SARS2** | 0.91 | 0.99 | > 0.05 | NA |
| *Fusobacterium* | 9.76E-07 | 0.49 | 0.043 | **SARS2** | 2.81E-08 | 0.3 | 0.015 | **FLUAV-SARS2** | 0.84 | 0.98 | > 0.05 | NA |
| *Haemophilus* | 1.58E-08 | 0.50 | 0.043 | **SARS2** | 6.23E-14 | 0.30 | 0.015 | **FLUAV-SARS2** | 0.61 | 0.89 | > 0.05 | NA |
| *Moraxella* | 0.00088 | 0.51 | 0.043 | **SARS2** | 0.78 | 0.95 | > 0.05 | NA | 0.098 | 0.60 | > 0.05 | NA |

**S2 Table. Taxa considered significant among multiple differential analyses within the lungs.**

Shown in bold are enriched groups that were considered significant in at least 2 of the 3 differential analyses performed. Adjusted p-values are reported: Deseq2 (Benjamini-Hochberg adjusted p-value) and ALDEx2 (Benjamini-Hochberg adjusted p-value using Wilcox t-test). p < 0.05 was considered significant for ALDEx2 and LefSE analysis and p < 0.01 was considered significant for Deseq2 analysis.
