# Supplementary material for: Pathobiology and dysbiosis of the respiratory and intestinal microbiota in 14 months old Golden Syrian hamsters infected with SARS-CoV-2
Source: PLoS Pathog. 2022 Oct 24;18(10):e1010734. doi: 10.1371/journal.ppat.1010734 (PMC9632924; doi:10.1371/journal.ppat.1010734)
Supplement: S3 Table — Pre-Challenge group was not included within these analyses. Shown in bold are enriched groups that were considered significant in at least 2 of the 3 differential analyses performed. Adjusted p-values are reported: Deseq2 (Benjamini-Hochberg adjusted p-value) and ALDEx2 (Benjamini-Hochberg adjusted p-value using Wilcox t-test). p < 0.05 was considered significant for ALDEx2 and LefSE analysis and p < 0.01 was considered significant for Deseq2 analysis. (DOCX) [file ppat.1010734.s013.docx]

**S3 Table.** **Taxa considered significant among multiple differential analyses of SARS-CoV-2 challenged aged Golden Syrian hamsters when samples from the intestine and feces were combined.**

|  | SARS2 v. Mock | | | | FLUAV-SARS2 v. Mock | | | | SARS2 v. FLUAV-SARS2 | | | |
| --- | --- | --- | --- | --- | --- | --- | --- | --- | --- | --- | --- | --- |
| **Taxa** | *Deseq2* | *ALDEx2* | *LefSE* | *Enriched* | *Deseq2* | *ALDEx2* | *LefSE* | *Enriched* | *Deseq2* | *ALDEx2* | *LefSE* | *Enriched* |
| Unclassified Bifidobacteriaceae | 0.95 | 0.88 | > 0.05 | NA | 0.0034 | 0.34 | > 0.05 | NA | 1.7E-04 | 0.16 | 0.0058 | **SARS2** |
| *Parabacteroides* | 0.021 | 0.20 | 0.036 | SARS2 | 0.0026 | 0.11 | 0.0084 | **FLUAV-SARS2** | 0.76 | 0.86 | > 0.05 | NA |
| Unclassified Prevotellaceae | 3.8E-33 | 8.4E-04 | 4.2E-05 | **Mock** | 1.1E-17 | 4.7E-04 | 1.8E-04 | **Mock** | 0.52 | 0.81 | > 0.05 | NA |
| *Elusimicrobium* | 3.0E-07 | 0.037 | 0.0028 | **SARS2** | 4.7E-07 | 0.0060 | 2.4E-04 | **FLUAV-SARS2** | 0.68 | 0.92 | > 0.05 | NA |
| *Fibrobacter* | 9.1E-09 | 0.21 | > 0.05 | Mock | 0.75 | 0.28 | > 0.05 | NA | 1.7E-04 | 3.7E-05 | 5.1E-06 | **FLUAV-SARS2** |
| *Allobaculum* | 0.33 | 0.34 | 0.036 | NA | 0.0025 | 0.034 | 7.2E-04 | **Mock** | 0.067 | 0.60 | 0.033 | NA |
| *Anaerostipes* | 2.2E-07 | 0.34 | 0.017 | **SARS2** | 0.69 | 0.86 | 0.027 | FLUAV-SARS2 | 3.4E-15 | 0.36 | > 0.05 | SARS2 |
| *Christensenellaceae R-7 group* | 0.044 | 0.38 | 0.045 | SARS2 | 6.4E-05 | 0.94 | 0.0060 | **FLUAV-SARS2** | 0.85 | 0.77 | > 0.05 | NA |
| *Ligilactobacillus* | 1.0E-07 | 0.14 | 0.0048 | **Mock** | 5.9E-05 | 0.023 | 7.6E-04 | **Mock** | 0.92 | 0.75 | > 0.05 | NA |
| *Mycoplasma* | 4.5E-05 | 0.07 | 0.0059 | Mock | 0.46 | 0.17 | > 0.05 | NA | 0.25 | 0.67 | > 0.05 | NA |
| *UCG-005* | 0.44 | 0.29 | > 0.05 | NA | 6.3E-07 | 0.034 | 0.0065 | **FLUAV-SARS2** | 1.4E-08 | 0.25 | 0.024 | **FLUAV-SARS2** |
| Unclassified Eubacteriaceae | 0.064 | 0.029 | 1.3E-04 | **SARS2** | 0.85 | 0.040 | > 0.05 | NA | 0.029 | 0.59 | 0.0068 | SARS2 |
| Unclassified Lachnospiraceae | 9.1E-05 | 0.34 | > 0.05 | Mock | 3.5E-08 | 0.19 | 0.041 | **Mock** | 0.26 | 0.84 | > 0.05 | NA |
| Coxiella | 0.21 | 0.35 | > 0.05 | NA | 0.040 | 0.26 | > 0.05 | FLUAV-SARS2 | 1.7E-04 | 0.0088 | > 0.05 | **FLUAV-SARS2** |
| Unclassified Spirochaetaceae | 5.2E-05 | 0.038 | 0.0015 | **SARS2** | 2.1E-15 | 9.4E-04 | 5.5E-06 | **FLUAV-SARS2** | 0.75 | 0.62 | > 0.05 | NA |

Pre-Challenge group was not included within these analyses. Shown in bold are enriched groups that were considered significant in at least 2 of the 3 differential analyses performed. Adjusted p-values are reported: Deseq2 (Benjamini-Hochberg adjusted p-value) and ALDEx2 (Benjamini-Hochberg adjusted p-value using Wilcox t-test). p < 0.05 was considered significant for ALDEx2 and LefSE analysis and p < 0.01 was considered significant for Deseq2 analysis.
