# Supplementary material for: Pathobiology and dysbiosis of the respiratory and intestinal microbiota in 14 months old Golden Syrian hamsters infected with SARS-CoV-2
Source: PLoS Pathog. 2022 Oct 24;18(10):e1010734. doi: 10.1371/journal.ppat.1010734 (PMC9632924; doi:10.1371/journal.ppat.1010734)
Supplement: S4 Table — Shown in bold are enriched groups that were considered significant in at least 2 of the 3 differential analyses performed. Adjusted p-values are reported: Deseq2 (Benjamini-Hochberg adjusted p-value) and ALDEx2 (Benjamini-Hochberg adjusted p-value using Wilcox t-test). p < 0.05 was considered significant for ALDEx2 and LefSE analysis and p < 0.01 was considered significant for Deseq2 analysis. (DOCX) [file ppat.1010734.s014.docx]

**S4 Table.** **Taxa considered significant among multiple differential analyses within the different anatomical sites in the intestine and feces.**

|  | SARS2 v. Mock | | | | FLUAV-SARS2 v. Mock | | | | SARS2 v. FLUAV-SARS2 | | | |
| --- | --- | --- | --- | --- | --- | --- | --- | --- | --- | --- | --- | --- |
| **Taxa** | *Deseq2* | *ALDEx2* | *LefSE* | *Enriched* | *Deseq2* | *ALDEx2* | *LefSE* | *Enriched* | *Deseq2* | *ALDEx2* | *LefSE* | *Enriched* |
| Small Intestine (Duodenum and Ileum) | | | | | | | | | | | | |
| Unclassified Bifidobacteriaceae | 0.96 | 0.95 | > 0.05 | NA | 0.035 | 0.57 | > 0.05 | Mock | 0.0099 | 0.31 | 0.027 | **SARS2** |
| Prevotella | 0.034 | 0.64 | > 0.05 | SARS2 | 0.0022 | 0.11 | 0.005 | **FLUAV-SARS2** | 0.44 | 0.67 | > 0.05 | NA |
| Unclassified Prevotellaceae | 9.47E-06 | 0.63 | 0.040 | **Mock** | 8.22E-08 | 0.44 | > 0.05 | Mock | NA | 0.82 | > 0.05 | NA |
| *Fibrobacter* | NA | 0.85 | > 0.05 | NA | 0.0072 | 0.45 | 0.019 | **FLUAV-SARS2** | 0.0099 | 0.47 | 0.037 | **FLUAV-SARS2** |
| *Allobaculum* | 0.24 | 0.50 | > 0.05 | NA | 0.0040 | 0.1 | 0.0050 | **Mock** | 0.033 | 0.30 | 0.011 | SARS2 |
| *Mycoplasma* | 0.008 | 0.18 | 0.0065 | **Mock** | 0.45 | 0.45 | > 0.05 | NA | 0.037 | 0.72 | > 0.05 | FLUAV-SARS2 |
| Unclassified [Eubacterium] coprostanoligenes group | 0.035 | 0.85 | > 0.05 | SARS2 | 0.93 | 0.94 | > 0.05 | NA | 0.0099 | 0.46 | 0.0075 | **FLUAV-SARS2** |
| Unclassified Spirochaetaceae | 0.45 | 0.89 | > 0.05 | NA | 0.004 | 0.27 | 0.0026 | **FLUAV-SARS2** | 0.0099 | 0.22 | 0.0097 | **FLUAV-SARS2** |
| Cecum | | | | | | | | | | | | |
| Unclassified Prevotellaceae | 1.4E-10 | 0.52 | 0.011 | **Mock** | 7.2E-05 | 0.45 | 0.039 | **Mock** | 0.74 | 0.94 | > 0.05 | NA |
| *Fibrobacter* | 0.0077 | 0.66 | > 0.05 | NA | 0.98 | 0.97 | > 0.05 | NA | 1.8E-05 | 0.11 | 0.0033 | **FLUAV-SARS2** |
| *Allobaculum* | 0.0077 | 0.53 | 0.02 | **Mock** | 0.0089 | 0.28 | 0.017 | **Mock** | 0.98 | 0.99 | > 0.05 | NA |
| *UCG-005* | 0.45 | 0.85 | > 0.05 | NA | 0.010 | 0.27 | 0.030 | **FLUAV-SARS2** | 0.036 | 0.61 | > 0.05 | **FLUAV-SARS2** |
| Unclassified Spirochaetaceae | 1.5E-09 | 0.59 | > 0.05 | SARS2 | 1.2E-09 | 0.29 | 0.016 | **FLUAV-SARS2** | 0.98 | 1 | > 0.05 | NA |
| Feces | | | | | | | | | | | | |
| *Parabacteroides* | 0.036 | 0.2 | > 0.05 | SARS2 | 2.6E-04 | 0.038 | 0.0078 | **FLUAV-SARS2** | 0.75 | 0.81 | > 0.05 | NA |
| Unclassified Prevotellaceae | 4.8E-24 | 0.034 | 0.0018 | **Mock** | 5.7E-12 | 0.014 | 8.7E-04 | **Mock** | 0.61 | 0.86 | > 0.05 | NA |
| *Elusimicrobium* | 4.5E-04 | 0.17 | 0.027 | **SARS2** | 6.5E-05 | 0.047 | 0.0077 | **FLUAV-SARS2** | 0.96 | 0.98 | > 0.05 | NA |
| *Fibrobracter* | 2.38E-04 | 0.21 | > 0.05 | NA | 0.83 | 0.89 | > 0.05 | NA | 1.85E-07 | 0.049 | 4.6E-04 | **FLUAV-SARS2** |
| *Ligilactobacillus* | 0.0053 | 0.28 | 0.027 | **Mock** | 7.8E-07 | 0.16 | 0.016 | **Mock** | 0.25 | 0.78 | > 0.05 | NA |
| *Anaerostipes* | 2.4E-04 | 0.44 | > 0.05 | SARS2 | 0.97 | 0.73 | > 0.05 | NA | 3.5E-08 | 0.4 | 0.047 | **SARS2** |
| *Christensenellaceae R-7 group* | 0.019 | 0.17 | 0.022 | SARS2 | 0.0013 | 0.22 | 0.015 | **FLUAV-SARS2** | 0.37 | 0.92 | > 0.05 | NA |
| *NK4A214 group* | 0.026 | 0.59 | > 0.05 | SARS2 | 1.70E-05 | 0.094 | 0.011 | **FLUAV-SARS2** | 0.89 | 0.73 | > 0.05 | NA |
| *UCG-005* | 0.95 | 0.44 | > 0.05 | NA | 0.0019 | 0.17 | 0.02 | **FLUAV-SARS2** | 4.3E-09 | 0.36 | 0.02 | **FLUAV-SARS2** |
| Unclassified Lachnospiraceae | 2.3E-04 | 0.12 | 0.0063 | **Mock** | 1.4E-06 | 0.015 | 0.0018 | **Mock** | 0.59 | 0.79 | > 0.05 | NA |
| Unclassified Spirochaetaceae | 3.7E-04 | 0.062 | 0.0078 | **SARS2** | 1.36E-06 | 0.14 | 0.0097 | **FLUAV-SARS2** | 0.26 | 0.79 | > 0.05 | NA |

Shown in bold are enriched groups that were considered significant in at least 2 of the 3 differential analyses performed. Adjusted p-values are reported: Deseq2 (Benjamini-Hochberg adjusted p-value) and ALDEx2 (Benjamini-Hochberg adjusted p-value using Wilcox t-test). p < 0.05 was considered significant for ALDEx2 and LefSE analysis and p < 0.01 was considered significant for Deseq2 analysis.
