# Supplementary material for: Pathobiology and dysbiosis of the respiratory and intestinal microbiota in 14 months old Golden Syrian hamsters infected with SARS-CoV-2
Source: PLoS Pathog. 2022 Oct 24;18(10):e1010734. doi: 10.1371/journal.ppat.1010734 (PMC9632924; doi:10.1371/journal.ppat.1010734)
Supplement: S5 Table — Shown in bold are enriched groups that were considered significant in at least 2 of the 3 differential analyses performed. Adjusted p-values are reported: Deseq2 (Benjamini-Hochberg adjusted p-value) and ALDEx2 (Benjamini-Hochberg adjusted p-value using Wilcox t-test). p < 0.05 was considered significant for ALDEx2 and LefSE analysis and p < 0.01 was considered significant for Deseq2 analysis. (DOCX) [file ppat.1010734.s015.docx]

**S5 Table.** **Taxa considered significant among multiple differential analyses comparing FLUAV pre-exposure to the mock pre-challenge and post-challenged fecal samples.**

|  | FLUAV Pre-challenge v. Mock Pre-challenge | | | | SARS2 v. Pre-challenge | | | | FLUAV-SARS2 v. Pre-challenge | | | |
| --- | --- | --- | --- | --- | --- | --- | --- | --- | --- | --- | --- | --- |
| **Genus** | *Deseq2* | *ALDEx2* | *LefSE* | *Enriched* | *Deseq2* | *ALDEx2* | *LefSE* | *Enriched* | *Deseq2* | *ALDEx2* | *LefSE* | *Enriched* |
| *Bifidobacterium* | 0.92 | 0.96 | > 0.05 | NA | 2.7E-04 | 0.09 | 0.0095 | **SARS2** | 0.035 | 0.45 | 0.033 | NA |
| *Prevotella* | 0.75 | 0.96 | > 0.05 | NA | 0.0080 | 0.15 | 0.045 | **Pre-Challenged** | 0.92 | 0.55 | > 0.05 | NA |
| *Allobaculum* | 0.99 | 0.96 | > 0.05 | NA | 0.0028 | 0.11 | 0.025 | **SARS2** | 0.55 | 0.63 | > 0.05 | NA |
| *Ileibacterium* | 0.92 | 0.99 | > 0.05 | NA | 4.9E-05 | 0.055 | 0.0032 | **SARS2** | 0.026 | 0.21 | 0.0057 | FLUAV-SARS2 |
| *Faecalibacterium* | 0.99 | NA | > 0.05 | NA | 3.8E-05 | 0.022 | 0.0015 | **SARS2** | 0.026 | 0.19 | 0.0043 | FLUAV-SARS2 |
| Anaerostipes | 5.3E-09 | 0.18 | 0.0015 | **Mock** | 0.80 | 0.65 | >0.05 | NA | 0.0047 | 0.82 | > 0.05 | Pre-challenge |
| Unclassified Eubacteriaceae | 0.71 | 0.98 | >0.05 | NA | 2.3E-07 | 0.017 | >0.05 | **SARS2** | 0.28 | 0.53 | 0.021 | FLUAV-SARS2 |

Shown in bold are enriched groups that were considered significant in at least 2 of the 3 differential analyses performed. Adjusted p-values are reported: Deseq2 (Benjamini-Hochberg adjusted p-value) and ALDEx2 (Benjamini-Hochberg adjusted p-value using Wilcox t-test). p < 0.05 was considered significant for ALDEx2 and LefSE analysis and p < 0.01 was considered significant for Deseq2 analysis.
