# Supplementary material for: Pathobiology and dysbiosis of the respiratory and intestinal microbiota in 14 months old Golden Syrian hamsters infected with SARS-CoV-2
Source: PLoS Pathog. 2022 Oct 24;18(10):e1010734. doi: 10.1371/journal.ppat.1010734 (PMC9632924; doi:10.1371/journal.ppat.1010734)
Supplement: Attachment — Submitted filename: 20220920_ResponsetoReviewers_PPathogens.docx [file ppat.1010734.s016.docx]

Manuscript PPATHOGENS-D-22-01212

Response to Reviewers

Dear Editors and Reviewers,

Thank you for giving us the opportunity to submit a revised manuscript of “Pathobiology and dysbiosis of the respiratory and intestinal microbiota in 14 months old Golden Syrian hamsters infected with SARS-CoV-2” for Plos Pathogens. We appreciate yours along with the reviewers’ time and effort spent providing feedback and insightful comments to help improve our manuscript. We have incorporated additional analyses and the suggestions/comments by the reviewers. Those changes are highlighted within the manuscript. Please see below a point-by-point response to the reviewers’ comments and concerns. Line numbers refer to the revised manuscript file with tracked changes.

**Part II – Major Issues: Key Experiments Required for Acceptance**

**Reviewer #1**

Page 3, lines 94–95. The authors mention that “To analyze the pathobiology, host response and effects of SARS2 and pre-exposure of influenza A virus……………”. Nevertheless, they infected the 14-month-old hamsters with an influenza A virus strain (i.e., A/Puerto Rico/8/34 (H1N1)), which does not replicate in this animal model. In fact, they did not detect antibodies post-A/Puerto Rico/08/34 (H1N1) inoculation. A previous study indicates that Syrian hamsters are highly susceptible to influenza A/H1N1 2009 pandemic viruses (Iwatsuki-Horimoto et al., JV, 2018). Such influenza A virus strains that can replicate well in the respiratory tracts of Syrian hamsters should be used.

**Author Response:** We appreciate the feedback from reviewer 1. While other influenza A virus strains replicate well in Syrian hamsters, information on the replication capacity of A/Puerto Rico/08/34 (H1N1) was unknown in the Syrian hamster model but is highly adapted in a different rodent species (mice) (1). Further, a previous paper also showed HI antibody titers in 2-3 month old Syrian hamsters three weeks after immunization with 200 chick cell agglutinating units of A/Puerto Rico/08/34 (H1N1) vaccine (2). Therefore, we decided to infect the Syrian hamsters with A/Puerto Rico/08/34 (H1N1) virus strain for this study.

**References**

1.	Pan W, Dong Z, Li F, Meng W, Feng L, Niu X, Li C, Luo Q, Li Z, Sun C, Chen L. 2013. Visualizing influenza virus infection in living mice. Nat Commun 4:2369.

2.	Jennings R, Brand CM, McLaren C, Shepherd L, Potter C. 1974. The immune response of hamsters to purified haemagglutinins and whole influenza virus vaccines following live influenza virus infection. Med Microbiol Immunol 160:295-309.

**Reviewer #2:**

1. No antibody could be detected in the treated mice. Can the authors confirm that the influenza challenge has any immunological impact in the hamsters? Is hamster susceptible to PR8?

**Author Response:** We appreciate reviewer 2 feedback. We cannot confirm that the influenza challenge has any immunological impact as samples were not collected for analysis on the immune response post influenza infection. Regarding susceptibility, a previous paper showed that 2–3-month-old Syrian hamster have detectable HI antibody titers post vaccination of 200 chick cell agglutinating units of A/Puerto Rico/08/34 (H1N1) (1).

**References**

1. Jennings R, Brand CM, McLaren C, Shepherd L, Potter C. 1974. The immune response of hamsters to purified haemagglutinins and whole influenza virus vaccines following live influenza virus infection. Med Microbiol Immunol 160:295-309.

2. It is unclear why the authors assume the findings observed from the FLUAV-SARS2 is entirely due to innate responses. Can the authors exclude the potential effects from T cell responses?

**Author Response:** We thank reviewer 2 for pointing this out. We cannot exclude potential effects from T cell responses as this was not analyzed within this study. Line 131-133 was modified to state: “Therefore, the effects, if any, of prior-FLUAV exposure in GSH challenged with SARS2 would likely not be related to stimulation of antibody responses.”

3. This manuscript is highly descriptive. Many of them are not really so important or directly related to the microbiota results (e.g. most histopathology results between SARS and FLUAV-SARS2 are very similar). The reviewer has difficulties to link their findings. This reviewer finds the summary from some supplementary tables are much easier to follow.

**Author Response:** We appreciate reviewer 2 feedback. We reduced the pathology descriptions within the text (line 169-319) and only included the respiratory tissue panels, which shows the largest differences among the groups, within the main text (Fig. 2). We also included the histopathological scores for the respiratory tissues within the main text (Table 1). The intestinal tissue panels, which do not show as much difference, were moved to a supplemental figure (S2 Fig) and the corresponding scores remained in Supplemental Table 1.

4. Following the same line, it will be more useful to elaborate their findings from the microbiota analysis to the observation related to disease severity, or even the clinical observation found in humans.

**Author Response:** Thank you for the reviewer’s suggestion. We discussed the relationship among the lung microbiota changes and disease severity by performing Spearman correlation analysis discussed in lines 484-507 and illustrated in Figure 4. We also performed the same analysis on the intestinal microbiota which is discussed in lines 611-630 and illustrated in Figure 6. Differential bacterial taxa were also related to clinical observations found in humans within the discussion.

5. Do RT-PCR positive tissues represent viremia or infected cells. Any data from immunological staining?

**Author Response:** Thank you for the reviewers’ comments. We included a supplemental figure (S1 Fig) for two respiratory tissues from the immunohistochemistry assay against SARS-CoV-2 virus antigen (red). We also added IHC results within the main text (lines 175-176, 290-292, 299-300) and details on the IHC methodology in the materials and methods section (lines 884-886).

6. Except the microbiota data from fecal sample collected at day -1 before SARS-CoV-2 infection, there is no data about the effect of FLUAV exposure on the diversity of microbiota. This might make the causal relationship between SARS-CoV-2 and microbiota less clear cut.

**Author Response:** We appreciate the reviewer’s input. We added additional results illustrating the analysis on the effect of FLUAV exposure on pre-challenged fecal samples. No significant differences were observed among the FLUAV pre-exposed and non-exposed hamsters within alpha diversity stated in lines 517-518 and graphed Fig S5B and beta diversity stated in lines 523-525 and graphed in Fig S6G-H. We also performed differential analysis and those results were stated in lines 606-609 and reported in Supplemental Table 5.

**Reviewer #3**

None

**Part III – Minor Issues: Editorial and Data Presentation Modifications**

Reviewer #1:

1. Page 3, lines 93–94. “we utilized an aged Syrian hamster model (14 months equivalent to ~60 human years)”. The authors need to cite reference(s) regarding correlation between hamster age and human age.

**Author Response:** Thank you for your suggestion. Due to the array of information about the equivalence of age, we removed these statements.

2. Fig. 2. The authors mention that “At 6-dpc, pneumonia is characterized by florid pneumocyte type II……. (Page 7, lines 189–190); However, up to 95% of the alveoli were lined by florid proliferation of cuboidal epithelial cells (pneumocytes type II), replacing the necrotic pneumocytes type I (Fig 2P-Q) (Page 8, lines 231–232)”. However, it is not clear whether the cells indicated by asterisks are type II pneumocytes to the reviewer. Higher magnification images of H&E staining of hamster’s organs should be included for a complete evaluation of the reader with regards to the extent of inflammation and histopathological changes represented at each time point.

**Author Response:** Thank you for the reviewer’s input. We added higher magnification (40X) inserts with details on the type 2 pneumocytes and inflammation in lines 208-210 and 293-296.

3. Fig.2. The authors need to provide the information on the magnification of the H&E image in the figure legend.

**Author Response:** Thank you. As per suggested by the reviewer, we added the magnification of the H&E images in the figure legend in lines 213 “*All H&E images are pictured at 20X magnification”.*

4. Page 21. How the A/Puerto Rico/8/34 (H1N1) virus was propagated should be included in the Experimental Procedures section.

**Author Response:** As per suggested by the reviewer, we added a description in lines 812-820 detailing the propagation and titration of A/Puerto Rico/8/34 (H1N1) virus.

5. Page 5, line 125. “HAI and ELISA” should be spelled out on the first use.

**Author Response:** As per suggested by the reviewer, we added the full spelling of HAI and ELISA assays in line 129-130.

Reviewer #2:

1. Line 125: Day 27 day the FIRST FLUVA dose?

**Author Response:** Thank you for pointing this out. The text was changed in line 129.

2. Supplementary tables: Please standardize terms “IAV-SARS2” and “FLUAV-SARS2” Or these are different models?

**Author Response:** Thank you for pointing this out. These are not different models, and the text was changed to FLUAV-SARS2.

3. SARS2 should be read as SARS-CoV-2. The former is a disease, not a a virus.

**Author Response:** Thank you. Within this manuscript SARS2 is an abbreviation for SARS-CoV-2.

Reviewer #3: The authors refer to modeling “geriatric patients” but GSH used here are equivalent to 60 y/o humans, 15 years younger than the currently accepted definition of “geriatric”. Suggest referring instead to modeling “older patients”.

**Author Response:** Thank you for the reviewers’ input. As per suggested, “geriatric” was changed to “older” patients when referring to the hamster model in line 96. However, we respectfully disagree as the definition for geriatric refers to an aged individual who requires special care. When referring to SARS-CoV-2, individuals over 60 years of age are at higher risk for disease severity needing special care for treatment.

The recent paper on SARS-CoV2 – FLUAV coinfection in hamsters (Oishi, et al, J. Virol. 2022) needs to be referenced and acknowledged in the Discussion.

**Author Response:** Thank you. As per suggested by the reviewer, a reference and comparison to Oishi et al. 2022 (J. Virol) is stated within the discussion in lines 661-663 and 721-723.
