# Supplementary material for: Pathobiology and dysbiosis of the respiratory and intestinal microbiota in 14 months old Golden Syrian hamsters infected with SARS-CoV-2
Source: PLoS Pathog. 2022 Oct 24;18(10):e1010734. doi: 10.1371/journal.ppat.1010734 (PMC9632924; doi:10.1371/journal.ppat.1010734)
Supplement: Attachment — Submitted filename: 202200930_ResponsetoEditors_PPathogens.docx [file ppat.1010734.s017.docx]

Manuscript PPATHOGENS-D-22-01212

Response to Reviewers

Dear Editors,

Thank you for giving us the opportunity to submit a revised manuscript of “Pathobiology and dysbiosis of the respiratory and intestinal microbiota in 14 months old Golden Syrian hamsters infected with SARS-CoV-2” for PLoS Pathogens. Notably, we have added textual revisions regarding the lack of confirmation of influenza A virus replication prior to SARS-CoV-2 challenge. Line numbers refer to the revised manuscript file with tracked changes.

Although most of the reviewers’ comments were addressed with revision, the lack of confirmation of IAV infection and viral replication in this model remains a concern. We request that the authors describe the evidence for a productive IAV infection in their animals, directly state whether this infection was confirmed or not in the results section, and to discuss any limitations to that data as a potential confounder in the manuscript.

While we do not have evidence of productive infection after FLUAV infection and, therefore, we are unable to confirm influenza A virus replication, we feel that the main focus of the study was to investigate the relationship of SARS-CoV-2 infection with age as a cofounding factor in a widely used animal model and the host residential microbiome. To directly address if influenza A virus replication was evaluated, we added a sentence within the results at lines 126-128 (reproduced here):

“Due to the limited number of the ~14-month-old hamsters, active FLUAV replication was not evaluated within this study.”

We also further discussed the lack of confirmation of influenza A virus replication within the limitations portion of the discussion in lines 609- 615 (reproduced here):

“Within this study, we did not evaluate FLUAV replication prior to SARS2 infection. However, FLUAV pre-exposure did have a distinct effect on the host microbial dynamics upon SARS2 infection compared to those that were only challenged with SARS2. Further research is needed to understand the impact of prior FLUAV infection and other respiratory pathogens and their role in stimulation of immune responses that might modulate outcomes in SARS2 infections due to effects on the residential respiratory and intestinal microbiota.”
